# A Healthy Diet Intervention Alters Food Preferences and Eating Behaviours Without Changing Appetite, Adipokines or Glucose Homoeostasis

**DOI:** 10.1155/jobe/8941754

**Published:** 2026-06-19

**Authors:** Litto Tharakan, Troy Merry, Eva Liu, Sara Cooper, Walter Hsu, Chris Hedges, Amber Parry-Strong, Fiona E. Lithander, Jeremy Krebs, Andrea Braakhuis

**Affiliations:** ^1^ Department of Nutrition, Faculty of Medical and Health Sciences, University of Auckland, Auckland, New Zealand, auckland.ac.nz; ^2^ Liggins Institute, University of Auckland, Auckland, New Zealand, auckland.ac.nz; ^3^ Centre for Endocrine, Diabetes and Obesity Research (CEDOR), Wellington, New Zealand; ^4^ Department of Medicine, University of Otago, Wellington, New Zealand, otago.ac.nz

**Keywords:** diet, food reward, implicit wanting

## Abstract

**Purpose:**

The study aimed to evaluate the influence of a 12‐week Mediterranean dietary pattern on eating behaviours, food preferences, appetite, satiety, hunger hormones, adipokines and glucose homoeostasis.

**Methods:**

This study is nested within the He Rourou Whai Painga (HRWP) trial. Adults with a high risk of metabolic disease (waist circumference–based metabolic score, MetSSS score ≥ 0.35) participated in the mixed‐meal challenge before and after a 12‐week dietary intervention. Questionnaire assessments included the Three Factor Eating Questionnaire (TFEQ) and the Leeds Food Preference Questionnaire (LFPQ). Visual analogue scales (VAS) were used to measure subjective appetite sensations. Blood was drawn in the fasting state and at 15‐, 30‐, 60‐, 90‐ and 120‐min postmeal. The following peptides, hormones and metabolic markers were analysed: adiponectin, ghrelin, leptin, peptide YY (PYY), glucagon‐like peptide‐1 (GLP‐1), gastric inhibitory polypeptide (GIP), fibroblast growth factor 21 (FGF21), growth differentiation factor 15 (GDF15), glucose, insulin and C‐peptide concentrations.

**Results:**

There was a significant decrease in uncontrolled eating (UE, *p* value = 0.0226) and an increase in cognitive restraint (CR, *p* value = 0.0004) following the intervention diet. Implicit desire for high‐fat savoury foods, low‐fat savoury foods and fat‐appeal bias was significantly reduced after the intervention diet. None of the peptides or metabolic markers changed significantly after the intervention diet. Even though glucose, insulin and C‐peptide levels decreased following the diet intervention, none of the values reached statistical significance.

**Conclusion:**

A healthy diet intervention alters food preferences and eating behaviours without changing appetite, adipokines or glucose homoeostasis.

**Trial Registration:** Australia New Zealand Trial Registry: ACTRN12622000906752

## 1. Introduction

It has been established that healthy dietary patterns, like the Mediterranean diet (MD), lower the risk of cardiovascular disease [1–6], prevent and treat metabolic illnesses [7], assist with weight maintenance [8], reduce postprandial desire to eat and improve satiety [9]. Populations that regularly consume a healthy dietary pattern have approximately 30% lower incidence of diabetes [10–12] and cardiovascular disease [3] and have been touted as an effective dietary strategy to reduce chronic disease [13]. Alongside the cardiovascular consequences of poor diet, weight gain and metabolic syndrome have been associated with increased appetite, a greater drive to consume food, altered gastrointestinal hormones and dysregulation of satiety and satiation mechanisms [14–16]. The mechanism or physiological change that explains improved metabolic health in those who follow a healthy diet may involve neurobiology and appetite‐related peptides that drive changes in eating behaviours.

In the context of our research, eating behaviour is shaped by our dieting mindset and encompasses our relationship with food. The Three‐Factor Eating Questionnaire (TFEQ‐R 18) is one tool that has been validated to assess eating behaviours within obesity and nutrition research [17]. The TFEQ has three distinct categories that represent uncontrolled eating (UE), emotional eating (EE) and cognitive restraint (CR) [18]. CR identifies strategic dieting behaviour, a general attitude of self‐control, and avoidance of unhealthy foods. While UE relates to excess hunger and inability to manage food portions, EE is linked to negative emotional states such as loneliness, depression or anxiety [19].

Food preferences are a distinct aspect of food behaviour and are driven by specific food and macronutrient factors. Food preference is an example of unconsciously learnt behaviour [20] and encompasses an individual’s degree of like or dislike for food and an assessment of its quality on a hedonic scale [21]. An example of a food preference is seeking high‐fat, high‐sugar foods due to taste, familiarity and a brain‐derived reward response [22]. Those who prefer highly palatable foods may be at an increased risk of metabolic disease [23], and higher ratings of liking for high‐fat foods have previously been linked to higher body fat levels [24]. Food preferences develop over time and can be influenced by cultural norms, food and nutrition knowledge, emotional associations with food and inherent food matrix, such as taste and texture [25]. There are certain biological variations in our perceptions of basic tastes, and these learning experiences develop over time and begin to shape as early as infancy [20, 26].

Food preferences can be influenced by the reward response elicited by food. The two aspects of food reward are pleasure, which is the enjoyable experience of eating, and food desire, which refers to the motivation or inclination to eat [27]. Food reward is a psychological phenomenon, and the mechanisms that regulate eating involve both unconscious (implicit) and conscious (explicit) processes [28]. Eating habits may not always be expressed explicitly. Explicit liking refers to the conscious and deliberate evaluation of how much one likes a particular food, often measured through self‐reported ratings or verbal expressions [29]. Implicit liking, on the other hand, involves automatic, unconscious preferences that can be assessed using indirect measures such as reaction times, physiological responses or behavioural tendencies. Explicit liking is typically influenced by cognitive processes and personal beliefs, while implicit liking is driven by more automatic, affective responses [29]. Understanding how food rewards are expressed, especially the ideas of liking and wanting, may be improved by this differentiation between implicit and explicit processes [30].

The psychological and physiological processes that drive our desire or craving for food (referred to as ‘food wanting’ or reward) might play a more significant role in weight gain and overeating than the actual choices or preferences we consciously make about what foods to eat. As such, the processes related to food ‘wanting’ or reward may be more relevant to weight gain and overconsumption than overt food choices or preferences [31]. Food desire is a stronger predictor of food consumption than food pleasure, highlighting its critical role in shaping eating behaviours and contributing to overconsumption [27]. In other words, the underlying urges and motivations to seek out and consume food, which can be influenced by factors such as stress, emotions and reward systems in the brain, could be more critical to overconsumption and weight gain than the foods we say we like or choose to eat. The biochemical parameters influence food preferences and regulate hunger and satiety. The anorexigenic hormones, such as glucagon‐like peptide‐1 (GLP‐1), peptide YY (PYY), the hunger hormone (ghrelin) and the adiposity signal (leptin), regulate appetite and satiety through complex neural interactions [32, 33]. The role of adiponectin is controversial, as it acts as both an appetite‐suppressing (anorexigenic) and an appetite‐stimulating (orexigenic) factor [34]. Glucose‐dependent insulinotropic polypeptide (GIP) plays a complex role in eating behaviour, primarily through its interaction with the central nervous system [35]. Fibroblast growth factor 21 (FGF21) is suggested to act as a compensatory signal to mitigate metabolic stress associated with obesity, and growth differentiation factor 15 (GDF15) is found to regulate energy expenditure in mice [36].

This study tested two aims: (1) to investigate whether a healthy dietary pattern of 12 weeks’ duration shifts the eating behaviours and food preferences in a population at risk for metabolic disease and (2) to investigate the postprandial appetite and satiety response. The secondary aim was to explore how the intervention diet altered appetite‐related peptides and glucose homoeostasis and whether these changes are associated with food preference and appetite. The implications of the findings will provide context for the effects of a healthy diet intervention concerning eating behaviours, preferences and appetite.

## 2. Materials and Methods

The data presented in this manuscript are from an experimental study nested within the wider He Rourou Whai Painga (HRWP) trial, a multicentre, randomised controlled trial in 200 index participants at risk of developing metabolic syndrome. The purpose of HRWP was to determine whether a MedDiet pattern that includes high‐quality foods from New Zealand (referred to as the ‘NZ MedDiet’) reduces the metabolic syndrome severity score (MetSSS) in people identified as at risk of cardiometabolic disease [37]. The primary investigation aimed to assess whether the New Zealand–modified Mediterranean diet pattern improved cardiometabolic health of people in Aotearoa New Zealand. The full protocol of the wider investigation has been published elsewhere [38], and details of the diet intervention have been characterised and published [37]. The primary outcome of the wider trial has been published [39].

### 2.1. Study Population

At the screening visit for HRWP, all participants completed a medical history, physical assessments (weight, waist circumference, height and blood pressure) and clinical assessments (lipids, HbA1C and glucose) and were informed about this substudy. Participants were adults aged 18–70 years with a MetSSS ≥ 0.35. Participants with preexisting Type 1 or Type 2 diabetes, blood pressure ≥ 180/100 mmHg, total cholesterol ≥ 8 mmol/L or those with severe food allergies were excluded from the study. Participants with end‐stage renal disease or gastrointestinal disorders such as inflammatory bowel disease or coeliac disease were also excluded. Participants were invited to participate in the current substudy procedures and told they would be participating in a study to investigate changes in mechanisms of human appetite and food preference before and after the intervention diet. All the participants were instructed about the procedures before providing written consent. Ethical approval was granted by the New Zealand Health and Disability Ethics Committee (Northern B branch), reference 2022 FULL 12045. The trial was registered with the Australia New Zealand Trial Registry.

### 2.2. Procedure

All participants were tested on two occasions, separated by 12 weeks (range ± 1 week). On the two test days, the participants arrived at the department at 9:00 h after an overnight fast (12 h). To complete the mixed‐meal tolerance test (MMTT), a participant was required to ingest a normal ‘mixed meal’, accounting for 25%–30% of their daily calorie intake orally. The participants’ weight, height, blood pressure, waist circumference and bioelectrical impedance analysis were measured before the MMTT. Visceral fat was measured using dual‐energy X‐ray absorptiometry (DEXA). The International Physical Activity Questionnaire (IPAQ) assessed physical activity before and after the intervention diet.

At 9:30 a.m., participants completed the Leeds Food Preference Questionnaire (LFPQ), appetite and hunger ratings and the TFEQ in the fasting state. Fasting venous blood samples were collected after cannulation. Breakfast was served at 9:45 a.m. The participants were requested to complete the questionnaires within 15 min. Venous blood samples are taken at various intervals throughout 120 min postprandial (15, 30, 60, 90 and 120 min).

### 2.3. Standardised Meal

A mixed‐meal challenge includes all macronutrients in a standardised format to induce a digestion and absorption challenge [40–42]. The meal consisted of toasted Signature Range English muffins (1 ½ splits), 13 g of Anchor butter, 22.5 g of Select raspberry jam spread on the muffins and 55 g of Complan chocolate‐flavoured powder mixed in water. The exact weight of each food item was carefully measured using electronic kitchen scales (energy: 2330 kJ, CHO: 71 g, fat: 23 g, protein: 19 g). Participants were required to consume the standardised meal within 10 min.

### 2.4. Eating Behaviours

The TFEQ‐R18 contains 18 questions related to eating behaviours, with participants asked to select the most appropriate response from five options. The questionnaire measures three aspects of eating behaviour—CR, UE, and EE—and has been validated for use in adult populations [18].

### 2.5. LFPQ

The LFPQ is a computerised behavioural task that measures ‘liking’ (pleasure/palatability) and ‘wanting’ (appetite/incentive motivation), the two physiological and functional components of food reward, using a 10‐min computer‐based association task [43]. The questionnaire contains two tasks. The first task requires participants to score 16 food photos arranged in random order. Questions are randomly counterbalanced rather than randomly generated. Participants were given standardised instructions to select the food they were most interested in eating. The order of tasks is either randomised or counterbalanced within the programme. The food pictures in the LFPQ are prevalidated such that the macronutrient content of the foods defines their categories (high fat: > 40% energy from fat; low fat: < 20% energy from fat), while matching protein content as closely as possible.

Food reward ratings were assessed twice for each subject, before and after identical breakfast meals (between 15 and 30 min), on two different occasions, using the LFPQ. This questionnaire was administered in a fasted state (before the standardised meal) and between the 15‐ and 30‐min blood test (fed state) at each visit. The online assessment was presented to participants using E‐Prime software (Version 3.0, Psychology Software Tools, Inc.).

The second task requires a quick choice between paired food combinations. The order of task completion is randomised. Implicit wanting was measured by a behaviour modification technique called ‘forced choice’. In the second task, 20 popular food images are employed and classified according to two criteria: fat content (high [HF] or low [LF]) and flavour (sweet [SW] or savoury [SA]). These foods are classified into four groups (HFSW, HFSA, LFSW or LFSA) or into four groups (HF, LF, SW and SA) based on their unique properties and combined features [43]. The meal that the participants ‘most want to eat’ must be chosen from two options during a forced‐choice exercise. Given that each item in the four food categories is paired with one stimulus from the other, there are 150 consecutive choices to be made. The task records each choice and sums the frequencies of choices for a particular food dimension to compare with those for other categories. The reaction time of each choice is recorded for use in the implicit wanting measure. Data from the forced‐choice exercise were stored on REDCap software. This included the frequency of each food choice and the response time for each choice.

### 2.6. Blood Collection and Analysis

Blood samples were withdrawn at 0, 15, 30, 60, 90 and 120 min following a standard meal. A 14‐gauge cannula was inserted, and blood was collected using a 10‐mL syringe (Vacutainer blood transfer device) at each time point after discarding a 1–2 mL draw. The blood samples were spun in a centrifuge (15 min at 2000 × *g*, 4°C), and the plasma samples were stored at −80°C until analysed. Glucose, insulin, C‐peptide and leptin were analysed from plasma (collected in an EDTA blood tube), while ghrelin, GLP‐1, GIP and PYY were also assessed from the plasma (collected in a BD P800 blood tube). Glucose, insulin and C‐peptide were analysed for all collected time points. Leptin, ghrelin, GLP‐1, GIP, PYY and adiponectin were analysed in plasma samples at fasting and at 30‐ and 120‐min postmeal. These time points are chosen to capture the dynamic postprandial responses of appetite‐related hormones and peptides [44]. Fasting provides a baseline value to account for the day‐to‐day variability of these peptides and hormones and isolate meal‐induced effects. A 30‐min time point is often used to capture the early postprandial peak or nadir concentrations of these biochemical markers. A 120‐min time point is used to observe the return of certain analytes to fasting or basal concentrations, as some appetite‐related peptides, such as ghrelin, GLP‐1 and PYY, may take longer to return to baseline levels [44].

The adipokines were analysed using an enzyme‐linked immunosorbent assay (ELISA) according to the kit manufacturer’s protocols. The absorbance was recorded spectrophotometrically over the wavelength range 450–590 nm after the reaction products were acidified, and the concentrations of the samples were calculated by interpolating from standard curves prepared with known amounts of the metabolic hormone tested. The ELIZA kit details are as follows: leptin (Human Leptin ELISA kit [ABCAM INC. ab108879‐1]), ghrelin (Human Ghrelin ELISA Kit [Ghrelin‐28, ABCAM INC. ab263887‐1]), GLP‐1 (Human GLP‐1 [1‐37a] ELISA Kit [Merck Life Science Ltd. (New Zealand) EZGLP1T‐36K]), GIP (Human GIP ELISA Kit [Merck Pty Limited 900000011423]), PYY (Human PYY ELISA Kit, ABCAM INC. ab255727‐1), adiponectin (adiponectin‐coated ELISA 96T Kit Thermo Fisher Scientific New Zealand Ltd. BMS2032‐2), FGF21 (Human FGF21 ELISA kit [EZHFGF21]) and GDF15 (ELISA kit RAB0204‐1KT).

Glucose, insulin and C‐peptide concentrations were analysed using a Cobas c 311 analyser (Roche Diagnostics, Mannheim, Germany).

### 2.7. Hunger and Satiety Visual Analogue Scales (VAS)

Appetite status was assessed at each visit using an online VAS questionnaire (Qualtrics; SAP), validated for single‐meal investigations [45]. The VAS was used to measure hunger, satiety, prospective food consumption and the palatability of the meals (desire to eat something fatty, salty, sweet or savoury). First, participants responded to the question, ‘How pleasurable would it be to taste this food right now?’ In addition to answering the questions ‘How hungry do you feel?’ (provides hunger scale) and ‘How full do you feel?’ (satiety scale), two more questions, such as ‘How much do you think you could eat right now? (Prospective Food Consumption Scale)’ and ‘How strong is your desire to eat right now? (Desire to Eat Scale)’, were filled. ‘Not hungry at all/full at all’ and ‘I am very hungry/full’ are anchored at each end. Subjects had to rate their subjective feelings on the four scales above. The participants used a 100‐unit sliding VAS to rate the enjoyment of tasting this food.

VAS hunger/satiety scores were recorded in the fasted state and 15, 30, 60, 90 and 120 min following the standard meal. Data were collected as values from 0 to 100 and converted to cm with 1 dp.

### 2.8. Statistical Analysis

The normality of the TFEQ data was assessed using the Shapiro–Wilk test and was deemed to follow a parametric distribution. The eating behaviours domain scores before and after the intervention diet were assessed using a paired *t*‐test. The food preference domains of implicit wanting, explicit liking and explicit wanting were all analysed using a 2‐way ANOVA. The overall appetite score was calculated using the following formula: overall appetite score = (satiety + fullness + [100 − hunger] + [100 − prospective food consumption])/4.

Glucose homoeostasis (glucose, insulin and C‐peptide), adiponectin, leptin, PYY, ghrelin, GLP‐1, GIP, FGF21 and GDF15; satiety, hunger, fullness, prospective food consumption and desire to eat (VAS scores) were all analysed at different time points before and after the intervention diet using a mixed‐effects ANOVA model to compare changes in outcome measures across time, with a Šidák correction for multiple comparisons. The area under the curve (AUC) was calculated for all biomarkers, including glucose, insulin and C‐peptide. The relationship between the eating behaviour domains and the AUC of all biomarkers was assessed using Pearson correlation (presented in supporting files, Table 1). Analysis and graphs were conducted using GraphPad Prism (Version 9.3.1).

## 3. Results

Eighty‐five adults expressed interest in participating. Of these, 21 participants entered the substudy: 10 from Auckland and 11 from Wellington. Two individuals were unable to attend the follow‐up visit after the intervention diet due to personal matters; therefore, results for 19 participants were available. Table 1 indicates the demographic characteristics of participants who performed the MMTT. Following the 12‐week healthy diet intervention, there was no change in the MetSSS; mean (SD) 0.01 (±0.0.6); weight decreased by mean (SD) 2.0 kg (±20), *p* = 0.0007; waist circumference was decreased by mean (SD) 3.0 cm (±0.14), *p* = 0.0117; and there was no significant change in visceral fat measured by DEXA, which was decreased by mean (SD) 44 gm (±1050 gm), *p* = 0.20.

**TABLE 1:** Baseline sociodemographic and anthropometrics of participants included in the analysis (*n* = 19).

Participant characteristics	Number	%
Gender		
Female	16	84
Male	3	16
Ethnicity		
New Zealand European	12	65
Māori	3	15
Indian	3	15
Samoan	1	5

	**Mean**	**SD**

Age (years)	49	13

	**Before the Med diet (Mean ± SD)**	**After the Med diet (Mean ± SD)**

MetSSS	1.13 ± 0.56	1.12 ± 0.58
Waist circumference (cm)	118 ± 14	115 ± 14^a^
Weight (kg)	102 ± 20	100 ± 20^a^
Visceral fat (g)	1410 1056	1366 ± 1054
Physical activity (minutes/day)		
Vigorous	1504 ± 877	1251 ± 1126
Moderate	1017 ± 1301	807 ± 497
Walk	582 ± 387	1435 ± 1269^a^
Total	1995 ± 1716	2695 ± 1983

^a^Indicates the that pre–post difference was statistically significant (*p* < 0.05).

**FIGURE 1:**
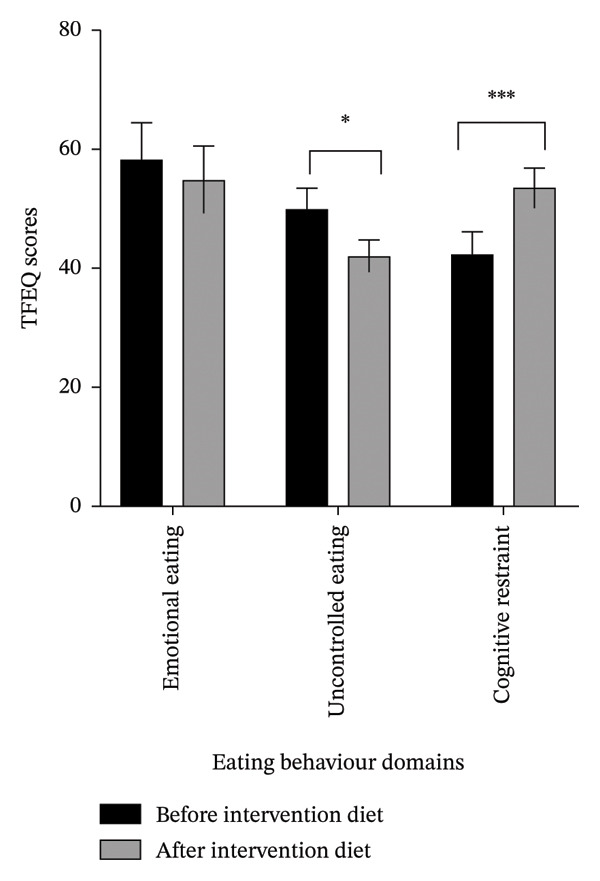
Three‐Factor Eating Questionnaire scores before and after the intervention diet. TFEQ = Three‐Factor Eating Questionnaire. Data are presented as mean (±SEM) = *p* < 0.05, (^∗∗^) = *p* < 0.0001.

Results of the eating behaviour questionnaire are shown in Figure 1. There was a significant decrease in UE and an increase in CR following the intervention diet. There was no significant change in the EE domain.

The participants’ food preferences and reward responses changed following the 12‐week dietary intervention. Fasted and fed scores before and after the diet intervention of the following food preferences, implicit wanting, explicit liking and explicit wanting of high‐fat savoury, low‐fat savoury, high‐fat sweet, low‐fat sweet, fat‐appeal bias and swee‐appeal bias are shown in Table 2. Food reward components, such as explicit liking, relate to how much the participant likes the food; implicit wanting reflects the automatic desire for the food and explicit wanting reflects the conscious desire for the food.

**TABLE 2:** Implicit wanting, explicit liking and explicit wanting for the food categories and appeal biases of the LFPQ on fasted and fed states before and after intervention diet.

Food dimension	Food reward component	Fasting		Fed		*p* value
		Before diet	After diet	Before diet	After diet	
High‐fat savoury	Explicit liking	46.73 (29.33)	36.50 (25.34)	32.30 (25.48)	25.84 (25.20)	0.64
High‐fat savoury	Implicit wanting	15.48 (24.07)	−2.99 (28.66)	5.12 (29.33)	1.71 (28.73)	0.015^∗^
High‐fat savoury	Explicit wanting	44.91 (29.45)	34.13 (27.04)	31.52 (26.85)	23.94 (28.29)	0.71
Low‐fat savoury	Explicit liking	48.15 (22.84)	44.89 (21.38)	32.62 (24.14)	32.98 (25.71)	0.609
Low‐fat savoury	Implicit wanting	2.40 (21.67)	13.67 (16.78)	4.24 (22.49)	4.32 (22.62)	0.027^∗^
Low‐fat savoury	Explicit wanting	49.01 (25.91)	46.73 (25.72)	30.31 (26.15)	31.16 (27.79)	0.708
High‐fat sweet	Explicit liking	35.68 (27.45)	34.46 (23.55)	28.52 (28.35)	23.89 (24.73)	0.471
High‐fat sweet	Implicit wanting	−25.7272 (31.28)	−18.80 (21.44)	−17.01 (31.81)	−14.75 (28.49)	0.25
High‐fat sweet	Explicit wanting	37.45 (30.17)	30.65 (25.57)	28.00 (29.77)	22.79 (27.88)	0.873
Low‐fat sweet	Explicit liking	50.16 (23.12)	46.06 (19.69)	36.61 (26.43)	34.63 (24.92)	0.756
Low‐fat sweet	Implicit wanting	7.85 (21.54)	8.12 (16.26)	7.65 (23.79)	8.71 (18.35)	0.954
Low‐fat sweet	Explicit wanting	52.61 (25.85)	46.83 (23.60)	36.12 (28.41)	33.51 (26.45)	0.674
Fat‐appeal bias	Explicit liking	−7.96 (14.17)	−9.99 (12.43)	−4.20 (11.54)	−8.94 (10.43)	0.453
Fat‐appeal bias	Implicit wanting	−10.25 (29.30)	−21.79 (25.34)	−11.89 (31.21)	−13.04 (28.60)	0.036^∗^
Fat‐appeal bias	Explicit wanting	−9.63 (15.58)	−14.39 (12.29)	−3.45 (11.50)	−8.98 (10.60)	0.881
Sweet‐appeal bias	Explicit liking	−4.52 (19.79)	−0.43 (11.72)	0.11 (12.02)	−0.15 (9.32)	0.187
Sweet‐appeal bias	Implicit wanting	−17.88 (30.59)	−10.68 (23.57)	−9.36 (32.60)	−6.03 (30.16)	0.481
Sweet‐appeal bias	Explicit wanting	−1.93 (18.96)	−1.69 (9.76)	1.14 (11.06)	0.60 (8.83)	0.776

*Note:* Data reported are mean ± SD. Food preference scores for the LFPQ before and after the intervention diet. *p* value includes the time‐by‐intervention interaction.

Abbreviation: LFPQ = Leeds Food Preference Questionnaire.

^∗^ *p* < 0.05.

Implicit wanting of high‐fat savoury showed a significant decrease after the diet in fasting, indicating a notable reduction in automatic desire for high‐fat savoury foods. Implicit wanting of low‐fat savoury foods increased significantly after the diet in fasting, suggesting a greater automatic desire for low‐fat savoury foods postdiet. Implicit wanting of the fat‐appeal bias significantly increased in fasting after the diet, indicating a stronger negative bias toward fat appeal postdiet.

The most notable changes occurred in implicit wanting, particularly for high‐fat savoury and low‐fat savoury foods, suggesting that the intervention diet may have altered automatic desires for these food types. Explicit liking and wanting generally remained stable across food categories, indicating that conscious preferences may not have shifted significantly despite changes in implicit desires.

The graphs are added as supporting data (See supporting Figures 1–3).

Given that participants reported reduced UE and increased CR following the intervention, we examined whether these dietary behavioural changes were reflected in perceived hunger and satiety responses before and after consuming a standardised meal. When VAS score responses were compared before and after the dietary intervention, no differences were observed in hunger, fullness, prospective food consumption or desire to eat. The AUC of the overall appetite score before the diet intervention was not significantly different after the diet intervention. The appetite suppression score showed no significant difference before versus after the intervention diet (see Figure 2).

FIGURE 2Subjective appetite scores measured on a VAS scale in response to the meal challenge before and after the intervention diet. The data presented are mean ± SEM. (a) Hunger, (b) fullness, (c) prospective food consumption and (d) desire to eat.

**Figure:**
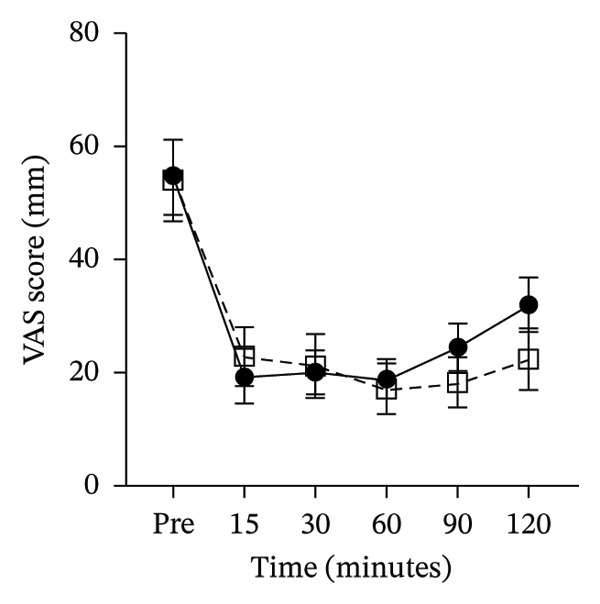
(a)

**Figure:**
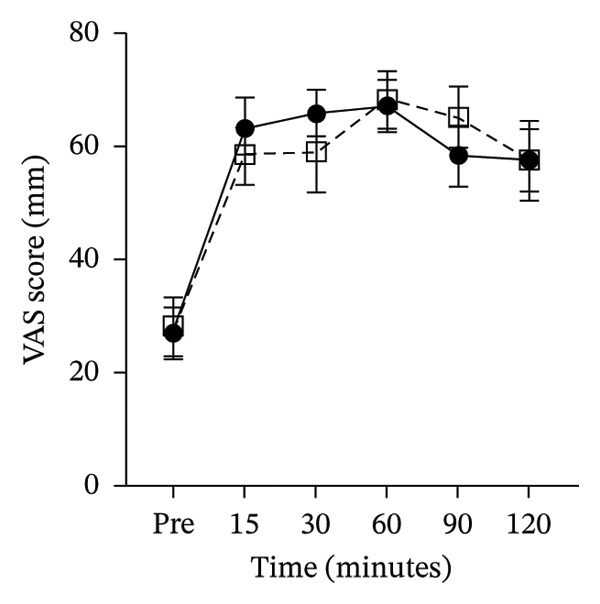
(b)

**Figure:**
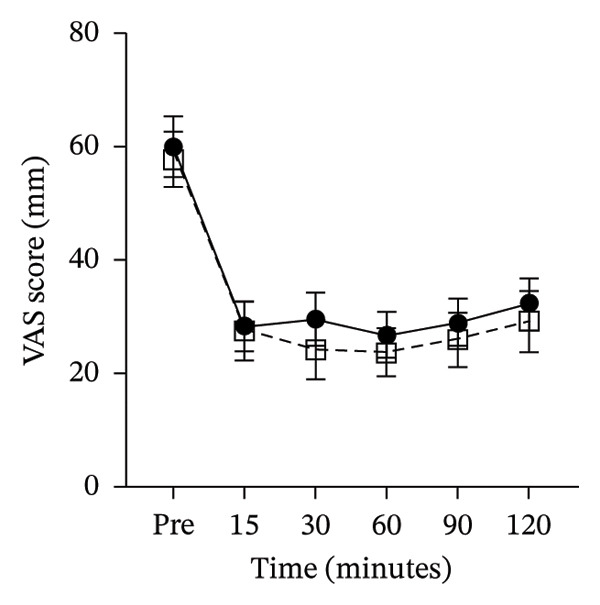
(c)

**Figure:**
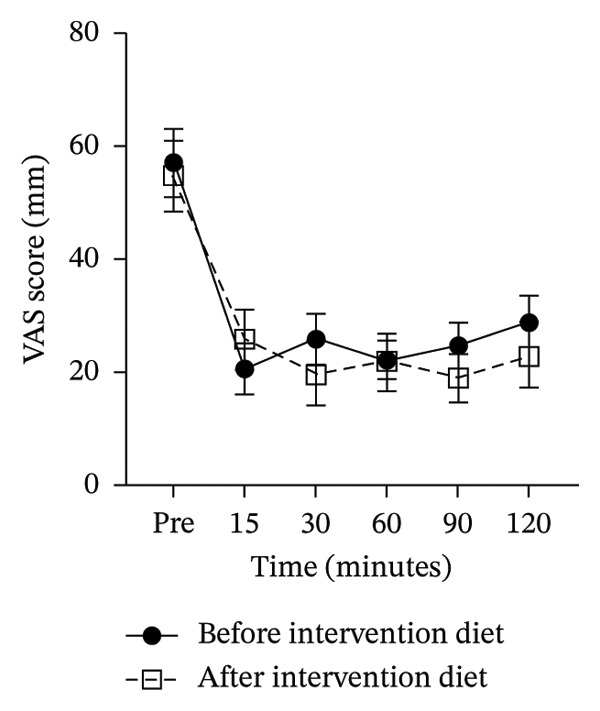
(d)

Adiponectin, leptin, PYY, ghrelin, GLP‐1, GIP, FGF21 and GDF15 were analysed in response to a mixed‐meal challenge before and after the intervention diet. None of the hormones or peptides showed a significant change due to the intervention diet. The changes in peptide levels (PYY, adiponectin, ghrelin, leptin, GIP, GLP‐1, FGF‐21 and GDF‐15) were not statistically significant after the intervention diet. Figures 3 and 4 display the experimental results showing the change in these hormone and peptide levels before and after the intervention diet. Individual hormone and peptide responses before and after the intervention have also been presented as supporting data (see supporting Figure 4), demonstrating that some individuals responded quite strongly to the intervention diet, while others responded less so. Unfortunately, the study is underpowered to investigate individual response rates in more detail.

FIGURE 3Changes in the hormones and peptides (adiponectin, leptin, PYY, ghrelin, GIP and GLP‐1) before and after the intervention diet. Data presented are mean ± SEM. (a) Adiponectin, (b) leptin, (c) PYY, (d) ghrelin, (e) GIP and (f) GLP‐1.

**Figure:**
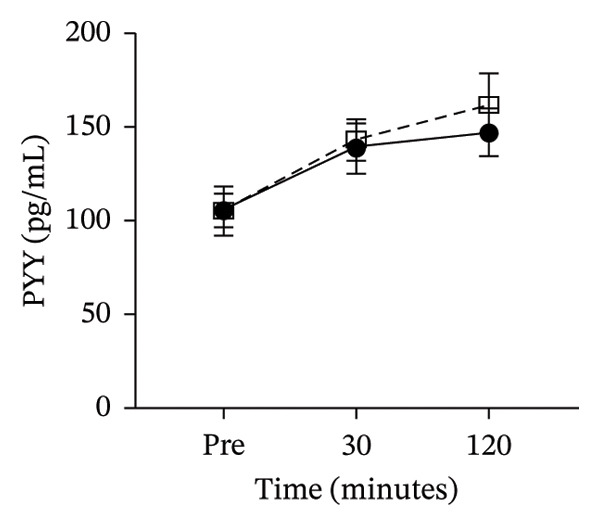
(a)

**Figure:**
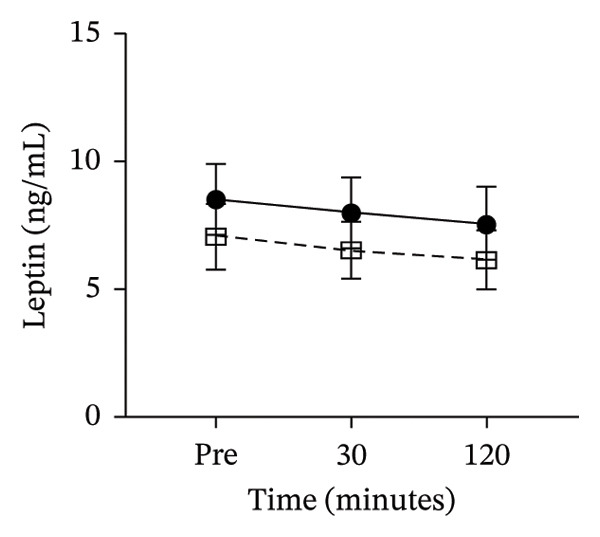
(b)

**Figure:**
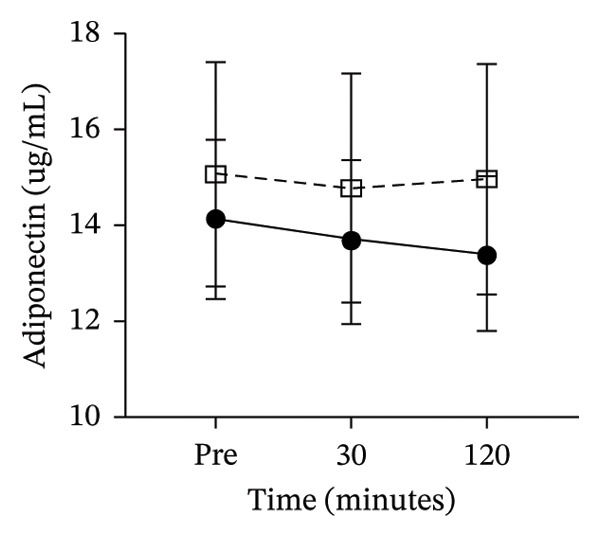
(c)

**Figure:**
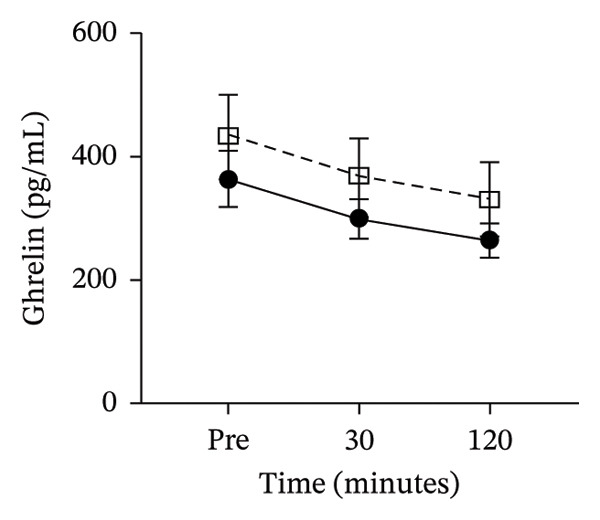
(d)

**Figure:**
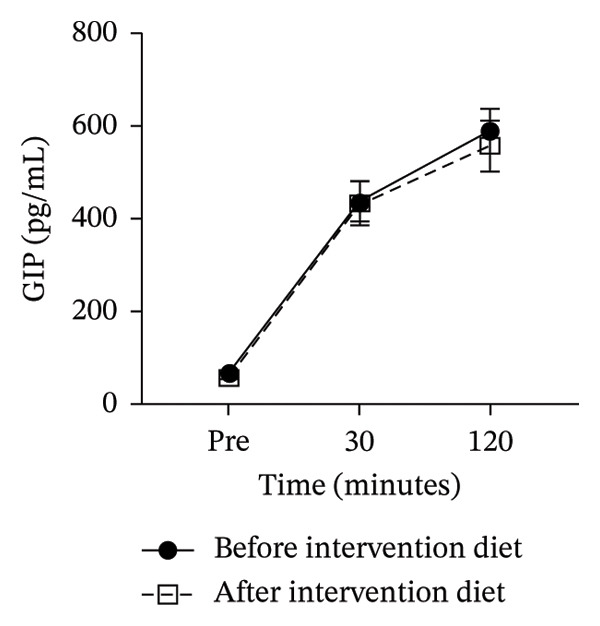
(e)

**Figure:**
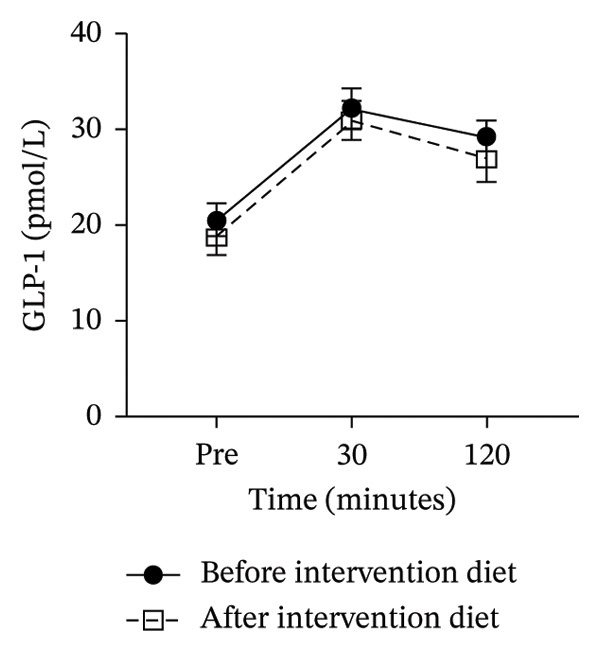
(f)

FIGURE 4Changes in FGF‐21 and GDF‐15 before and after the intervention diet. The data presented are the mean ± SEM (a) for FGF‐21 and (b) for GDF‐15.

**Figure:**
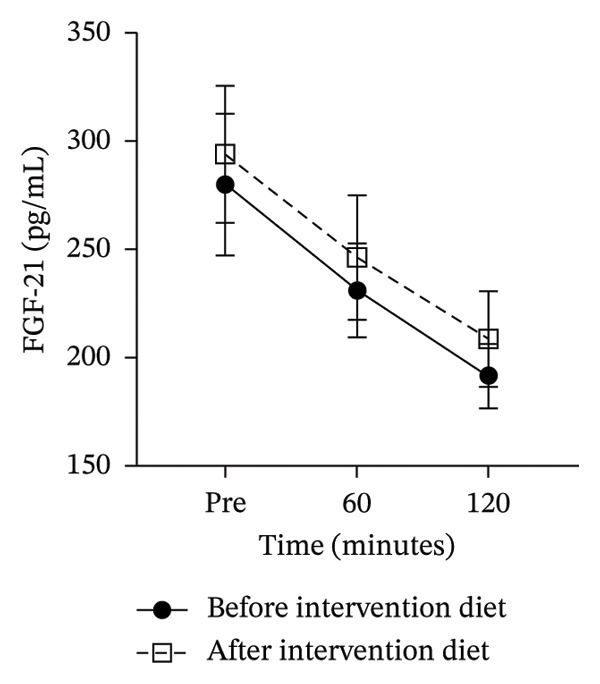
(a)

**Figure:**
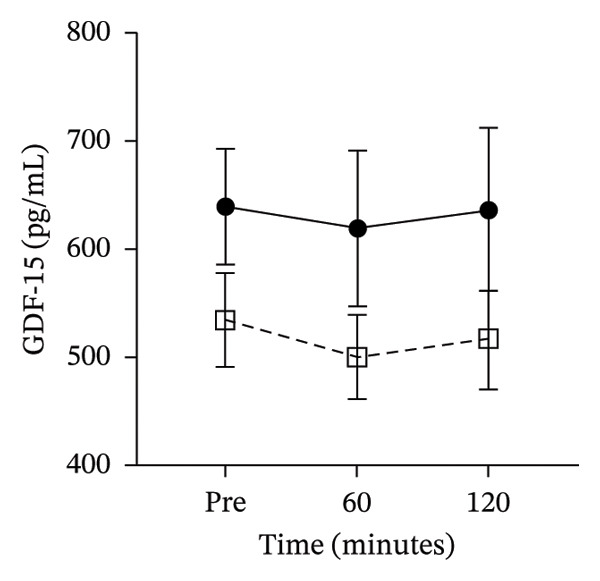
(b)

Glucose homoeostasis was assessed by measuring the glucose, insulin and C‐peptide response before and after the diet intervention. Different glycaemic control parameters are shown in Figure 5. Even though glucose, insulin and C‐peptide levels decreased following the diet intervention, none of the studies was statistically significant. AUC for glucose was as follows: before intervention diet: 38.5 ± 2.7 (95% CI 33–44); after intervention diet: 37.0 ± 2.5 (95% CI 32–42). AUC for insulin was as follows: before intervention diet: 695.9 ± 189.7 (95% CI 324–1068); after intervention diet: 615.2 ± 162 (95% CI 298–933). AUC for C‐peptide was as follows: before intervention diet: 52.2 ± 6 (95% CI 40–64); after intervention diet: 47.2 ± 5.7 (95% CI 36–58).

FIGURE 5Change in indices of glucose homoeostasis in response to a meal challenge before and after the intervention diet. Data presented are mean ± SEM. (a) Glucose, (b) insulin and (c) C‐peptide.

**Figure:**
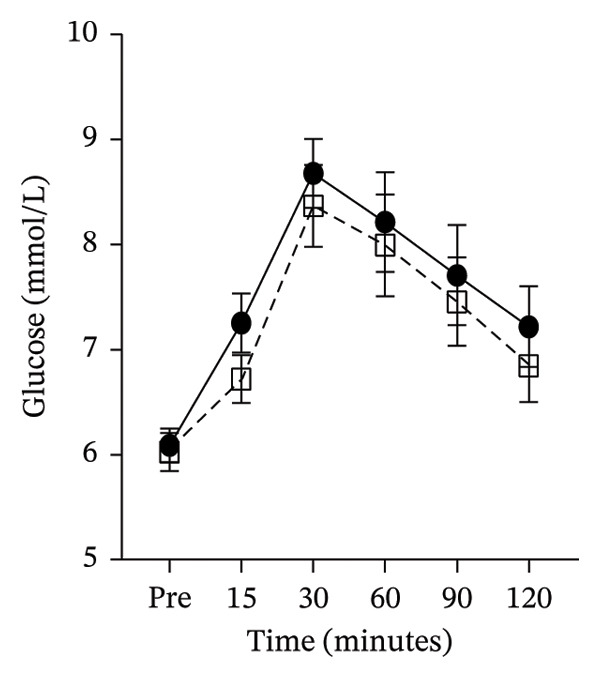
(a)

**Figure:**
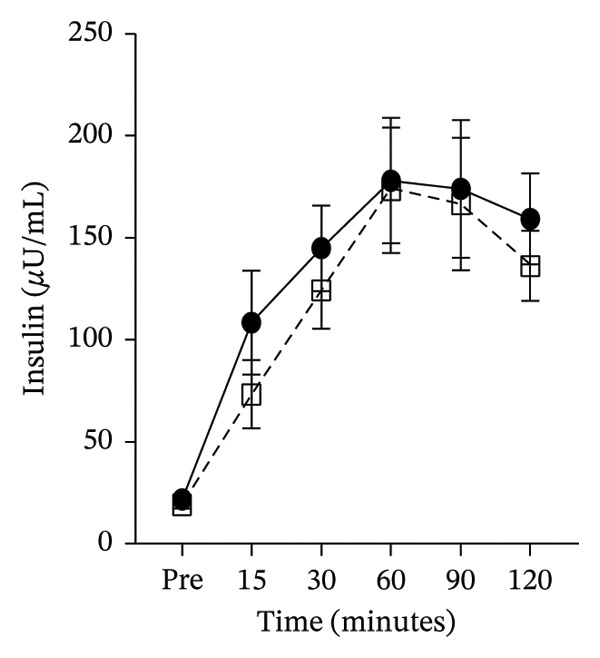
(b)

**Figure:**
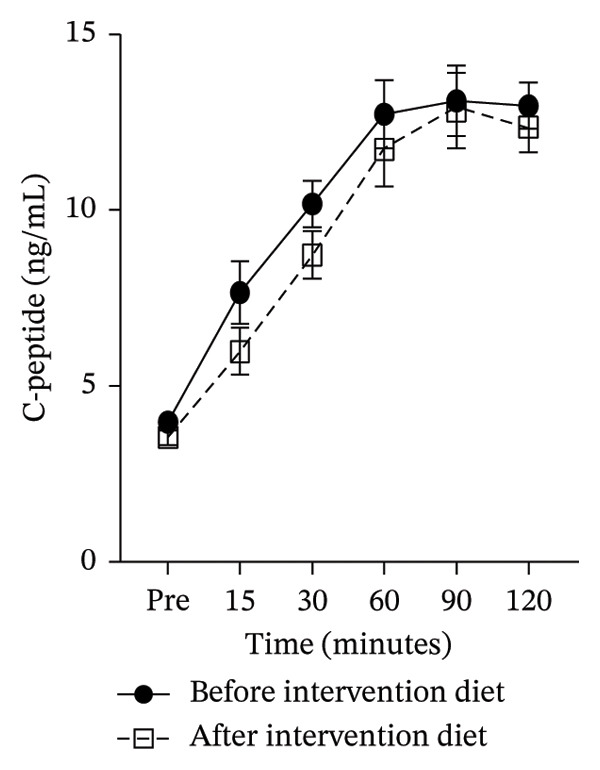
(c)

As supporting data, the correlation analysis between eating behaviour domains and the AUCs of different biomarkers revealed significant differences for FGF21 and GDF15. Significant correlation was found between EE and FGF21 (*r* = 0.66, *p* = 0.003). A significant negative correlation was also observed between CR and GDF15 (*r* = −0.45, *p* = 0.04).

## 4. Discussion

Participants at risk of cardiometabolic disease completed a MMTT before and after a 12‐week healthy diet intervention. We provide novel evidence of changes in eating behaviours and food preferences in the absence of changes in appetite or adipokine concentrations. The participants showed increased behaviours related to CR and lower UE, a shift in implicit wanting of food choices away from high‐fat toward low‐fat savoury foods and a decrease in the fat‐appeal bias. These changes in eating behaviours and preferences were independent of fasting and postprandial ratings of hunger and satiety, as well as plasma concentrations of appetite‐controlling hormones, including leptin, PYY, ghrelin, GLP‐1, GIP, adiponectin, FGF‐21 and GDF‐15.

It is well‐recognised that healthy diet interventions can reduce obesity and metabolic abnormalities, but how diet influences eating behaviours and vice versa is less clear [46]. According to a study conducted among college students, those who follow a healthy dietary pattern were more inclined to practice restrained eating [47]. In a group of postmenopausal women with obesity [48], those who reported improvements in their eating behaviours (such as reduced binge eating, EE and UE) lost more weight compared to those whose eating behaviours remained unchanged. Higher baseline restricted eating was also linked to greater weight loss in the postmenopausal women [48]. In our study, the participants’ body weight decreased by a mean of 2%, which, although statistically significant, is below the amount required to achieve clinically relevant improvements in metabolic health. As has been shown in previous successful weight‐loss programmes, decreases in body weight were also linked to changes in eating habits and food preferences [49–52]. As one of the domains of eating behaviours, CR refers to the conscious control over food intake, often to manage weight [53]. In previous research, an individual’s level of dietary CR has been shown to have a significant relationship with energy intake and body weight [50]. The combination of high dietary restraint and low UE has been associated with greater success in weight loss maintenance [50]. A meta‐analysis on the effect of various dietary interventions on EE suggested that these interventions have a delayed effect on EE compared to UE and CR [54].

A recently published study investigating the relationship between MD adherence and EE reported an inverse relationship between EE and MD adherence [55]. Although very low–carbohydrate ketogenic diets are found to be helpful in various eating disorders, the mechanism by which this diet influences food addiction disorders or binge eating is considered complex and multifactorial [56]. Our findings are consistent with past 12‐week healthy diet interventions, showing increased CR following a nutritional intervention. Since the shift in EE is too early to occur at 12 weeks, this increase in CR may be considered an early change in eating behaviour.

According to a study by Carbonneau et al. [49], a sample of 19 men with metabolic syndrome who followed a MD with notable weight loss was linked to decreased susceptibility to hunger, UE and greater CR [49]. In a related study conducted by Leblanc et al. [51], those on a MD showed greater CR and UE, which enhanced their capacity to control their weight and other improved health markers [51]. In the current study, we report similar changes in CR and EE but not hunger. In general, increased CR and lower UE may assist individuals in losing weight through behaviour modification [50, 52].

The LFPQ incorporates implicit measures, such as reaction‐time tasks related to food choice, to assess the motivational pull and the food reward associated with particular food options. [15]. Specifically, ‘wanting’ is the psychological motivation certain foods elicit, making them more desirable and prompting action to obtain them [57]. In the context of weight‐loss interventions, ‘wanting’ is associated with food reward and can help explain why some foods are more tempting and harder to resist than others. Obesity may be more related to increased motivation to eat rather than greater pleasure [31]. Implicit wanting incorporates the motivational aspects of reward‐seeking behaviour [15]. In dieting, you might ‘want’ a piece of cake even if you do not particularly ‘like’ it, due to its high motivational attraction [58]. In our study, following the intervention diet, implicit measures of food wanting reduced for high‐fat savoury foods and increased for low‐fat savoury foods. The implicit wanting related to fat‐appeal bias was also reduced. Our findings suggest that the dietary intervention significantly impacted the implicit desire for certain food types, particularly high‐fat savoury options, while explicit preferences remained stable.

A systematic review found that food reward, including liking and wanting high‐energy foods, generally decreases during weight management interventions [16]. In this review, dietary interventions were noted for their effectiveness, with four of five studies reporting changes in liking for high‐ or low‐energy foods, consistent with our findings. The change in implicit wanting in response to the dietary intervention is thought to be linked to reduced exposure, neuroadaptation and behavioural conditioning toward low‐fat savoury at the expense of high‐fat foods in response to the nutrition education and food provided in the dietary interventions [16].

Appetite is closely related to food reward and explicit food preferences. It is regulated by a complex interplay of hormones and neural signals that communicate hunger and satiety to the brain [59–61]. While satiety refers to the period between meals, hunger and propensity to eat have physiological and psychological mechanisms [15, 62, 63]. A study of university students concluded that explicit measures of food reward in the LFPQ are sensitive to hunger and correlate with one another, whereas implicit measures of the LFPQ do not show the same sensitivity or correlations [64]. In our study, the implicit wanting for high‐fat savoury food decreased, and the implicit wanting for low‐fat savoury food increased. Explicit measures of food reward did not alter significantly after the intervention diet, nor did the subjective appetite ratings.

Preferences for high‐fat and savoury foods, compared with low‐fat options, are partially associated with glucose regulation and appetite‐related peptides [65]. Disruptions in the interplay of these peptides can lead to metabolic dysfunction [66]. Ghrelin may act with brain reward systems to promote overeating of palatable foods [67]. Increased suppression of ghrelin levels following dietary intervention was associated with greater satiety [68]. The above study found that increased ghrelin suppression following dietary intervention was associated with greater satiety. A review on intervention diets and leptin concluded that leptin levels decreased significantly with energy‐restricted diets, potentially improving leptin sensitivity [69]. Leptin is known to regulate satiety, and leptin deficiency is associated with altered food preferences [70]. These anorectic factors are discussed as contributing to the transformation of a motivated food approach into anxiety‐driven avoidance, influencing eating behaviour and food preferences in individuals [70]. MD interventions have been found to significantly reduce leptin levels, though ghrelin and GLP‐1 levels showed variable effects [71].

Furthermore, it has been proposed that certain peptides or hormones may enhance the brain’s sensitivity to satiety signals, potentially resulting in decreased food intake and improved body weight management [72]. The gut hormones such as ghrelin, GLP‐1, leptin, adiponectin and GIP are important players in regulating appetite and satiety [65]. A study on leptin and restrained eating in binge‐eating disorder suggested that CR of food intake may lead to lower leptin production, rather than leptin influencing eating behaviour [73]. The intricate signalling pathways affected by high‐fat diets disrupt leptin and insulin signalling, which are essential for maintaining body weight and glucose homoeostasis [74]. A narrative review indicates that dietary interventions can effectively alter GLP‐1 levels and can contribute to better glycaemic control [75].

Food preferences and food reward responses are not only regulated by psychological processes but may also be linked to brain signalling via physiological messengers such as FGF21 and GDF15 [75]. Nutrient signalling and sensing are crucial components of the reward system that influence food preferences [60]. FGF21 has been shown to influence food choices and reduce cravings for sweet foods [76]. FGF21 acts as an endocrine signal that regulates metabolic homoeostasis by influencing energy expenditure and plays a critical role in macronutrient preferences, increasing protein intake [77]. In our study, EE was associated with FGF21 postintervention. FGF21 is considered a stress‐induced hormone that regulates energy homoeostasis and metabolism [78], and adherence to the MD is inversely associated with EE [55]. GDF15 plays a significant role in regulating food intake by acting as a potent anorectic factor [79]. High levels of GDF may shift preferences from energy‐dense foods. In our study, CR was negatively correlated with GDF15 at baseline in this cohort of individuals at risk of metabolic syndrome.

Despite the theoretical importance of appetite hormones and adipokines in eating behaviours and food preferences, we observed no differences in ghrelin, leptin, adiponectin or satiety signals, including GLP‐1, GIP, PYY, FGF‐21 and GDF‐15, after 12 weeks of the healthy intervention diet. Our intervention did not show significant differences in the adipokine meal response before or after the diet intervention, unlike reported previous research [68, 69, 80, 81].

### 4.1. Strength and Limitations

Our study provides novel evidence on the influence of a Mediterranean dietary pattern on eating behaviours, food preferences and metabolic markers. The strength of the study lies in its rigorous methodology, including a standardised MMTT and serial blood sampling to collect data. The use of validated questionnaires, such as the TFEQ to assess eating behaviours and the LFPQ to assess food preferences, further strengthens the study. Although these questionnaires may not capture the dynamic process of behaviour change, they were the validated questionnaires for the assessment. Diet monitoring using digital photography or a wearable device may be a more objective tool. However, in our population group, digital photography or other wearable devices were not validated, and the Otago Food Frequency Questionnaire was a more appropriate option.

However, there are a few limitations. The ability to detect physiological changes linked to eating behaviour may have been hampered by the small sample size, short intervention duration and a one‐shot 12‐week analysis, which could otherwise have had a greater impact. The small sample size limits the interpretability and generalisability of the findings. A mid‐term assessment at 6 weeks between the two time points (midway between the 12 weeks of intervention diet) and a follow‐up after 4 weeks of the intervention diet may be a more ideal design for precise nutrition. Such an assessment at specific time points might have helped to determine whether the behaviour change observed in the study is a short‐term adaptation or long‐term remodelling. Dietary assessment by self‐report may be subject to memory and social approval bias.

The eating behaviour phenotypes, such as the EE subtype, food addiction tendency and the circadian rhythm phenotypes (such as the early/late phenotype), were not included in the analysis. Leptin resistance in obese individuals, which may dilute the real physiological changes, was not accounted for in the analysis. A randomised controlled trial involving a larger number of participants and lasting for a longer duration, with questionnaire assessments every 4 weeks, would provide more robust evidence.

## 5. Conclusion

In a group of people at risk of cardiometabolic disease, the psychology of eating and food choice changed toward a profile that supports weight loss and improves eating behaviour following a Mediterranean dietary pattern intervention for 12 weeks; however, this was not reflected in the physiological biomarkers of eating and food choice.

## Author Contributions

Troy Merry and Andrea Braakhuis designed the research; Litto Tharakan, Eva Liu and Amber Parry‐Strong conducted the research; Litto Tharakan and Andrea Braakhuis conducted the statistical analysis; Andrea Braakhuis and Litto Tharakan wrote the manuscript; Andrea Braakhuis had primary responsibility for the final content of the manuscript; Jeremy Krebs was the primary investigator for the wider study. All authors provided content and feedback to the manuscript including Fiona E. Lithander and Jeremy Krebs.

## Funding

The authors declare that financial support was received for the research, authorship and/or publication of this article. High‐Value Nutrition National Science Challenge Funding supported this study through the NZ MBIE.

Open‐access publishing was facilitated by the University of Auckland, as part of the Wiley–the University of Auckland agreement via the Council of Australasian University Librarians.

## Disclosure

All authors read and approved the final manuscript.

## Conflicts of Interest

The authors declare no conflicts of interest.

## Supporting Information

Additional supporting information can be found online in the Supporting Information section.

## Supporting information


**Supporting Information** Supporting Table 1: Correlation analysis between eating behaviour domains and adipokines. Supporting Figure 1. Leeds Food Preference Questionnaire implicit wanting scores: Change before and after the diet intervention. Error bars indicate SEM. (^∗^) indicates significant change (*p* < 0.05). Supporting Figure 2. Leeds Food Preference Questionnaire explicit wanting scores: Change before and after the diet intervention. Error bars indicate SEM. Supporting Figure 3. Leeds Food Preference explicit liking scores: Change before and after the diet intervention. Error bars indicate SEM. Supporting Figure 4. Changes in the hormones and peptides (adiponectin, leptin, PYY, ghrelin, GIP and GLP‐1) before and after the intervention diet. The data presented are individual data points before and after the intervention. (A) Adiponectin, (B) leptin, (C) PYY, (D) ghrelin, (E) GIP and (F) GLP‐1.

## Data Availability

The data that support the findings of this study are available on request from the corresponding author. The data are not publicly available due to privacy or ethical restrictions.
