# Supplementary material for: A Healthy Diet Intervention Alters Food Preferences and Eating Behaviours Without Changing Appetite, Adipokines or Glucose Homoeostasis
Source: J Obes. 2026 Jun 19;2026:8941754. doi: 10.1155/jobe/8941754 (PMC13280808; doi:10.1155/jobe/8941754)
Supplement: Supplementary file 1 — Supporting Information Supporting Table 1: Correlation analysis between eating behaviour domains and adipokines. Supporting Figure 1. Leeds Food Preference Questionnaire implicit wanting scores: Change before and after the diet intervention. Error bars indicate SEM. (∗) indicates significant change (p < 0.05). Supporting Figure 2. Leeds Food Preference Questionnaire explicit wanting scores: Change before and after the diet intervention. Error bars indicate SEM. Supporting Figure 3. Leeds Food Preference explicit liking scores: Change before and after the diet intervention. Error bars indicate SEM. Supporting Figure 4. Changes in the hormones and peptides (adiponectin, leptin, PYY, ghrelin, GIP and GLP‐1) before and after the intervention diet. The data presented are individual data points before and after the intervention. (A) Adiponectin, (B) leptin, (C) PYY, (D) ghrelin, (E) GIP and (F) GLP‐1. [file JOBE-2026-8941754-s001.docx]

**Supplementary material**

The Leeds Food Preference Questionnaire is a computer-based behavioural task that measures the two functional components of food reward: explicit sensory pleasure or liking of food, or implicit motivation or wanting of food. The purpose of our study was to assess whether a 12-week Mediterranean dietary pattern would alter the food reward response in participants at risk of metabolic syndrome.

Following the 12-week dietary intervention, participants showed significant changes in food reward responses, particularly in implicit wanting. There was a notable decrease in the automatic desire for high-fat savoury foods and a substantial increase in the desire for low-fat savoury foods in the fasting state. Additionally, fat appeal bias increased, reflecting a stronger aversion to high-fat foods post-diet. In contrast, explicit liking and wanting remained largely unchanged, suggesting that while automatic food preferences shifted, conscious preferences were stable. Supplementary Figures 1–3 present the relevant data.

**Supplementary Table 1**: Correlation analysis between eating behaviour domains and adipokines.

|  | Emotional Eating | | Uncontrolled Eating | | Cognitive Restraint | |
| --- | --- | --- | --- | --- | --- | --- |
|  | Before diet | After diet | Before diet | After diet | Before diet | After diet |
| Leptin | -0.01 | 0.10 | 0.11 | 0.07 | 0.31 | 0.24 |
| Ghrelin | 0.31 | 0.33 | -0.04 | -0.14 | 0.18 | 0.27 |
| Adiponectin | 0.41 | 0.33 | -0.38 | -0.30 | -0.12 | -0.08 |
| GLP-1 | -0.07 | -0.1 | 0.13 | -0.48 | -0.04 | -0.23 |
| GIP | 0.23 | 0.32 | 0.07 | -0.30 | -0.02 | -0.29 |
| PYY | 0.09 | 0.13 | 0.07 | -0.11 | 0.39 | -0.03 |
| FGF21 | -0.05 | 0.66** | 0.06 | -0.39 | -0.13 | -0.35 |
| GDF15 | 0.44 | 0.04 | -0.37 | -0.17 | -0.45* | -0.36 |
| Footnote: Data reported as r=Pearson coefficient, ^**^ p=0.003; ^*^ p=0.04. | | | | | | |


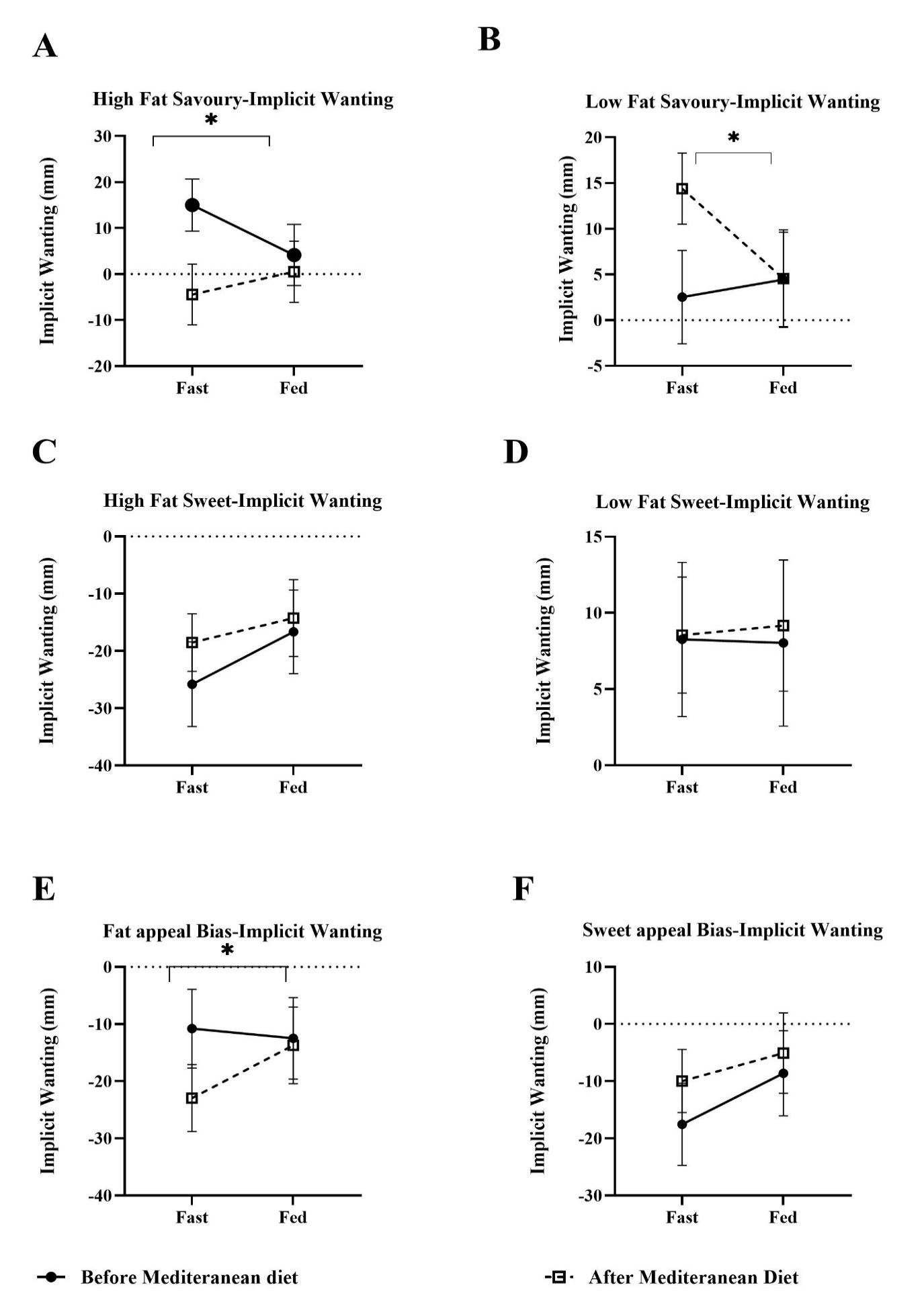


**Supplementary Figure 1**. Leeds Food Preference Questionnaire Implicit wanting scores: Change before and after the diet intervention. Error bars indicate SEM. (^*)^ Indicates significant change (p<0.05).


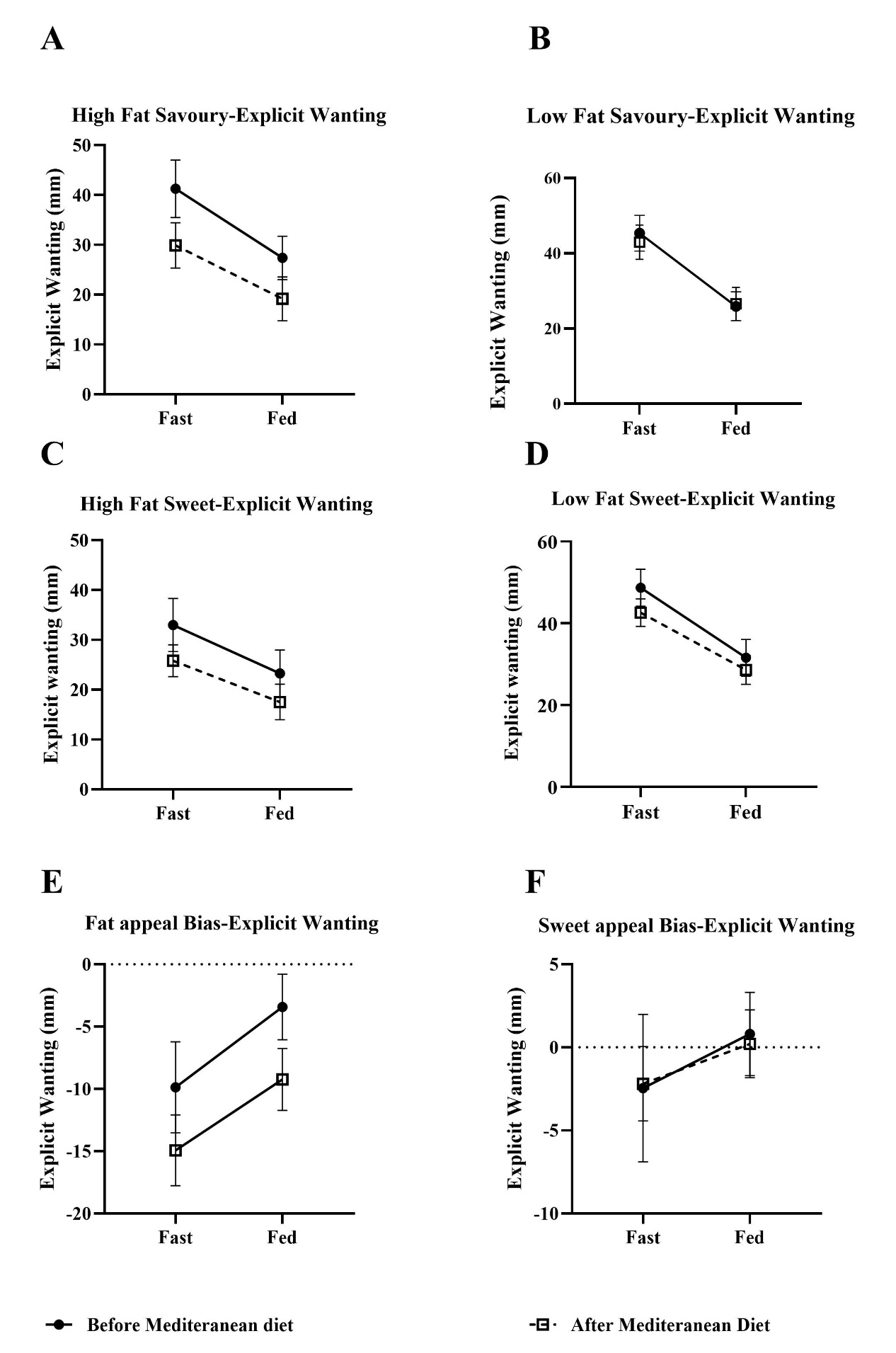


**Supplementary Figure 2.** Leeds Food Preference Questionnaire Explicit Wanting scores: Change before and after the diet intervention. Error bars indicate SEM.


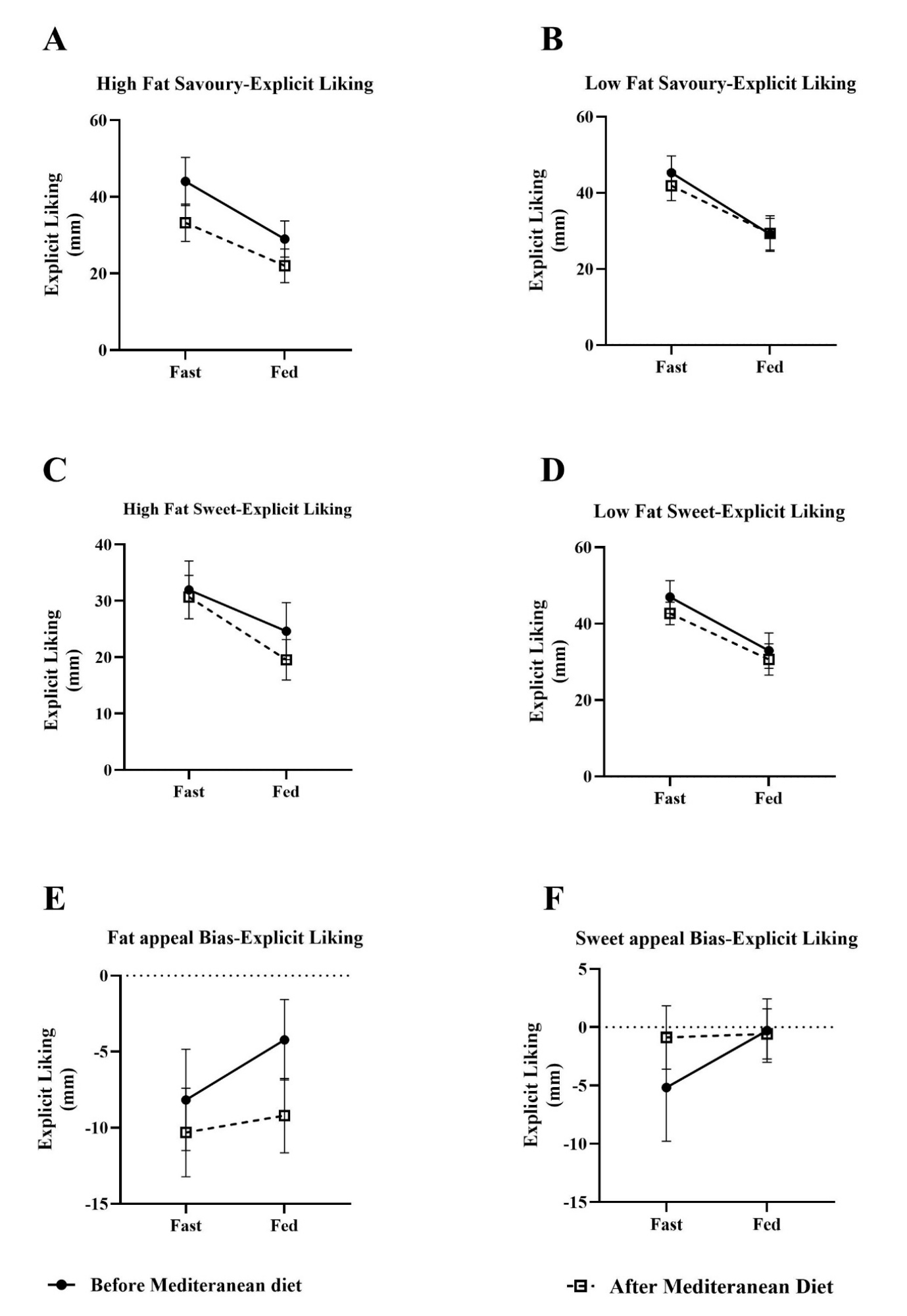


**Supplementary Figure 3.** Leeds Food Preference Explicit Liking scores: Change before and after the diet intervention. Error bars indicate SEM.


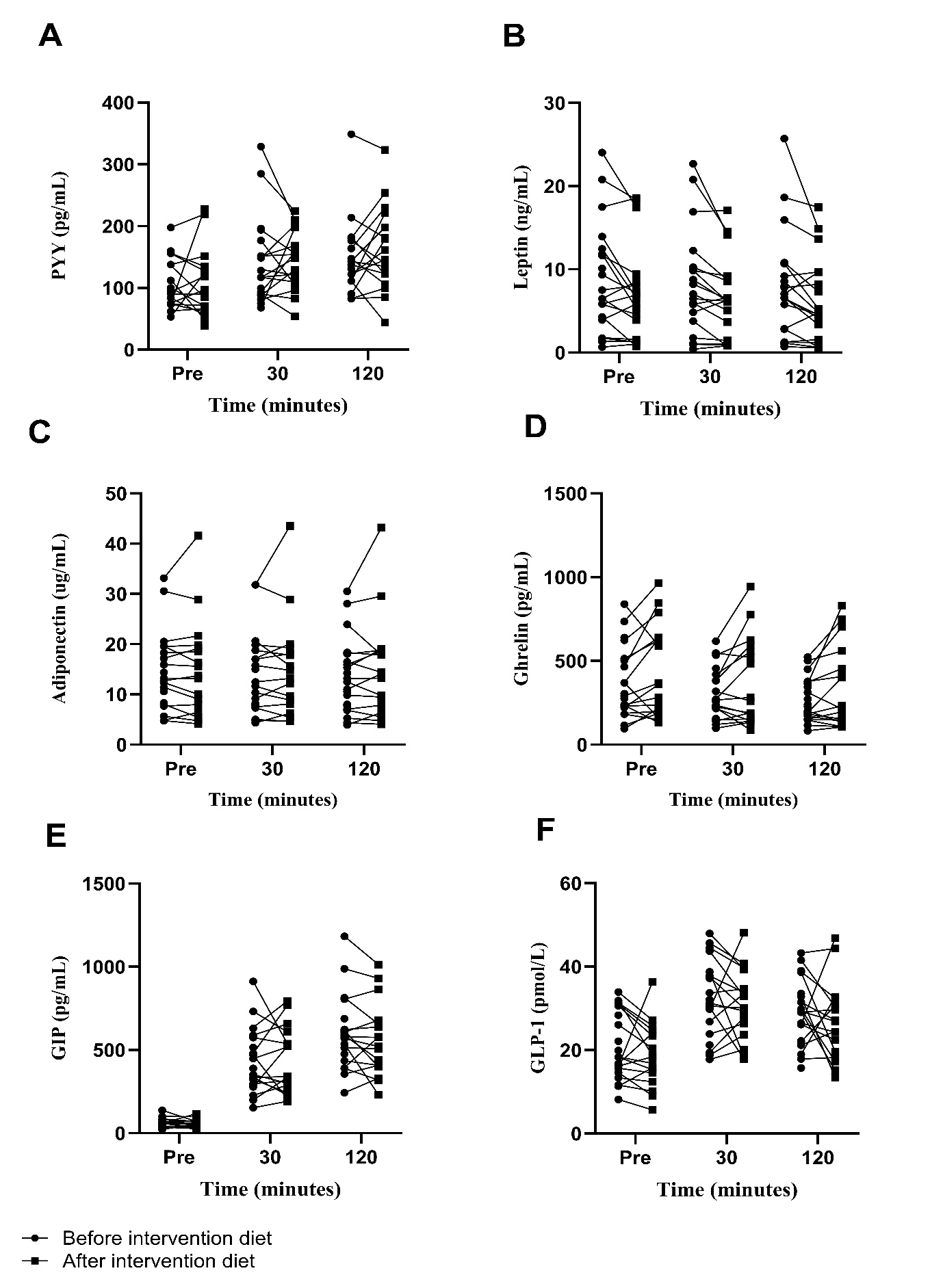


**Supplementary Figure 4.** Changes in the hormones and peptides (Adiponectin, Leptin, PYY, Ghrelin, GIP and GLP-1) before and after the intervention diet. The data presented are individual data points before and after the intervention. (A) Adiponectin, (B) Leptin, (C) PYY, (D) Ghrelin, (E) GIP, (F) GLP-1.
